# Retroperitoneal Chyloma: A Case Report and Literature Review

**DOI:** 10.7759/cureus.54924

**Published:** 2024-02-26

**Authors:** Norah I Alabdulaaly, Ahmed A AlAkeel, Raief F Alfriedy, Refah M Alajmi, Ashwag H AlHarbi, Mohammed AlJabali, Bandar A Idrees

**Affiliations:** 1 General Surgery, Prince Sultan Military Medical City, Riyadh, SAU; 2 Emergency Medicine, King Fahad University Hospital, Khobar, SAU; 3 General Surgery, Prince Sultan Military Medical City, Riyadh , SAU

**Keywords:** retroperitoneal chyloma, retroperitoneal, fat–fluid level, complete excision, chylous cyst, chyloma

## Abstract

Retroperitoneal chyloma is a rare entity that presents with non-specific symptoms. Although benign, it can cause complications due to the mass effect. In this case report, we present the case of a 24-year-old woman who presented with a complaint of left-sided colicky abdominal pain and mild dysuria for one year. On physical examination, there was only mild abdominal tenderness. Computed tomography (CT) revealed a thick-walled cystic retroperitoneal mass with a small amount of fat in the superior part and a displaced left hydronephrotic kidney. Magnetic resonance imaging (MRI) confirmed the findings and also revealed a fat-fluid level in the cyst. A laparotomy was performed, and the cystic mass, containing milky fluid, was excised. Histopathology showed a pseudocyst with chronic inflammation and a xanthomatous reaction, with no evidence of infection or malignancy. The patient recovered without complications and has not had a recurrence so far. Retroperitoneal chyloma is difficult to diagnose preoperatively. A definitive diagnosis is usually made only after surgery and a histopathological examination. The treatment of choice is a complete excision. Other approaches, such as marsupialization or drainage, will likely result in a recurrence. However, surgery in the retroperitoneal space is associated with a risk of injury to major vessels or organs. In conclusion, retroperitoneal chyloma is a rare entity that is best treated by complete excision. For small lesions, a wait-and-watch approach may be advisable.

## Introduction

Retroperitoneal cysts are rare with an estimated incidence of one in 5,750 to one in 250,000 people [1]. A small proportion of these are chylous cysts. Patients may be asymptomatic (about one-third) or may present with non-specific symptoms, making clinical diagnosis difficult. Imaging may be helpful, but surgery and pathological examination are usually necessary to confirm the diagnosis [1-3].

We report a case of retroperitoneal chyloma in a young woman and review the literature on this rare clinical entity. This work is consistent with the Surgical CAse REport (SCARE) criteria [4].

## Case presentation

A 24-year-old woman with a history of bronchial asthma presented with complaints of chronic abdominal pain for one year. The pain was on the left side, colicky in nature, and associated with mild dysuria, but there was no radiation. The pain had increased in severity over the past month, with no clear aggravating or relieving factors. There were no other gastrointestinal symptoms or history of weight loss. On examination, the patient looked well and was not dehydrated or in severe pain. Vital signs were normal. There was mild tenderness in the left upper quadrant of the abdomen and left flank, but no abdominal distention or signs of peritoneal irritation were noted.

**Figure 1:**
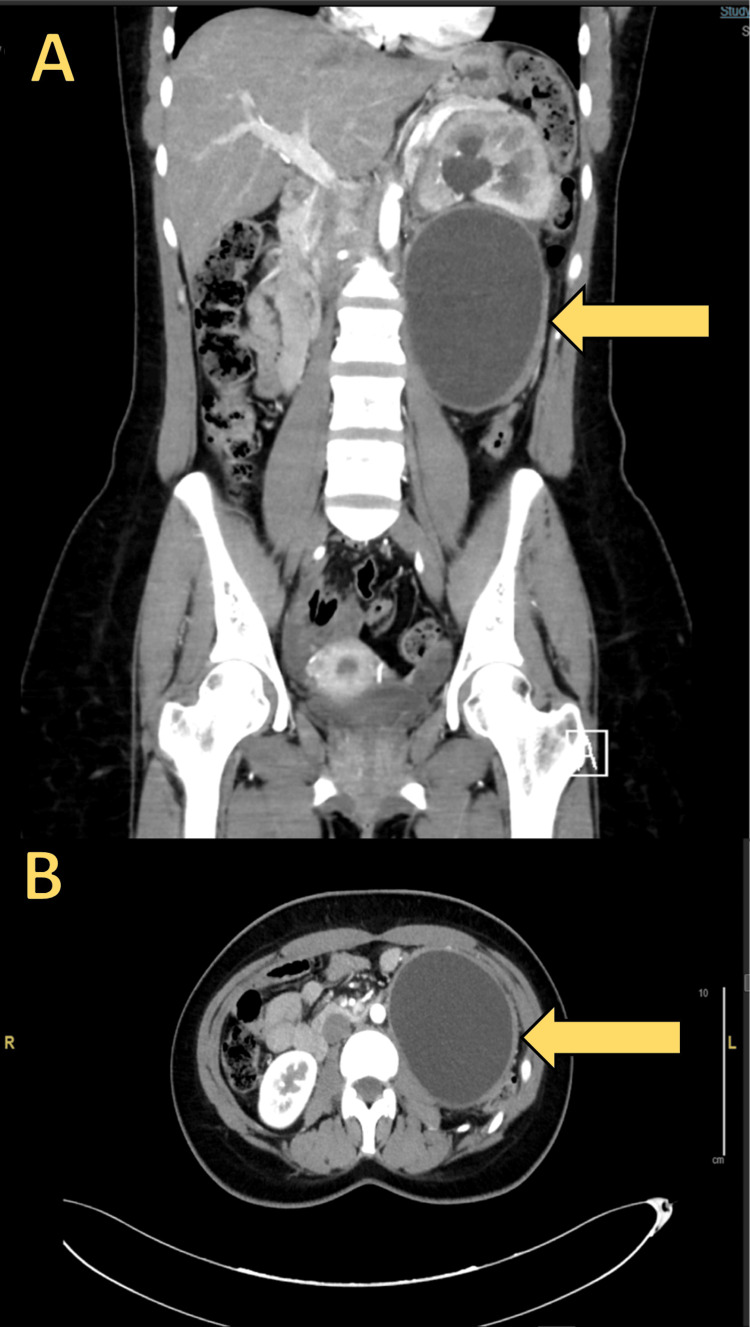
The CT scan shows a large, thick-walled cystic retroperitoneal mass containing a small amount of fat at the superior portion. (A) coronal plane of the abdominal CT; (b) transverse plane of the abdominal CT

Laboratory test results were unremarkable, tumor markers were within normal ranges, and a hydatid workup was negative. A culture of the fluid was aspirated from the cyst using a Veress needle connected to a 50-cc syringe. The aspirated fluid was negative for bacteria, acid-fast bacilli, and fungi. A plain abdominal radiograph was unremarkable, but computed tomography (CT) of the abdomen showed an 8 × 10 × 11 cm thick-walled cystic retroperitoneal mass containing a small amount of fat at the superior portion. There was no solid component. The mass had displaced the left kidney anteriorly and superiorly. The kidney showed grade 3 hydronephrosis and reduced parenchymal enhancement. A small wedge-shaped hypodensity was seen in the posterior aspect of the left kidney, suggestive of pyelonephritis or infarction. Medial to the hypodensity was a small pooling of contrast in the delayed phase, suggestive of early forniceal rupture. There was no evidence of contrast passage through the left ureter (Figure 1).

**Figure 2:**
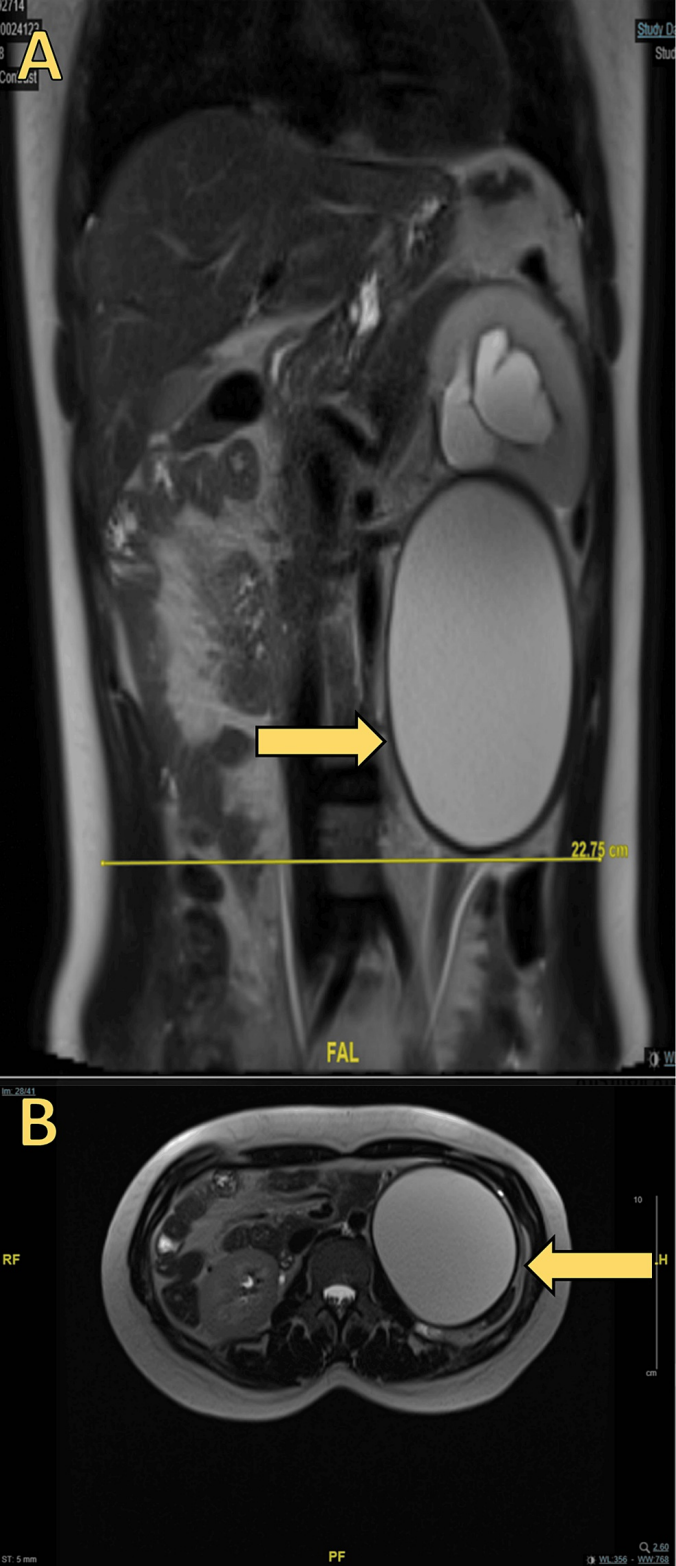
The MRI shows a T2-hypointense, thick-walled cystic retroperitoneal mass containing a small amount of fat at the superior portion and a fat-fluid level. (A) coronal plane of the abdominal MRI; (b) transverse plane of the abdominal MRI

Magnetic resonance imaging (MRI) showed a T2-hypointense, thick-walled cystic retroperitoneal mass containing a small amount of fat at the superior portion, with a fat-fluid level. The mass measured 9.3 × 8 × 10.7 cm and had no internal enhancing components, papillary projections, endocysts, or septations. The mass effect on the lower pole of the left kidney had caused moderate to severe left hydroureteronephrosis. The MRI confirmed the presence of the superior-posterior forniceal rupture as well as a urinoma measuring 1.1 × 2.5 × 2.4 cm (3 mL). The mass was clearly separate from the left kidney, pancreas, and adjacent muscular structures. There were no significantly enlarged abdominal or perilesional lymph nodes (Figure 2).

**Figure 3:**
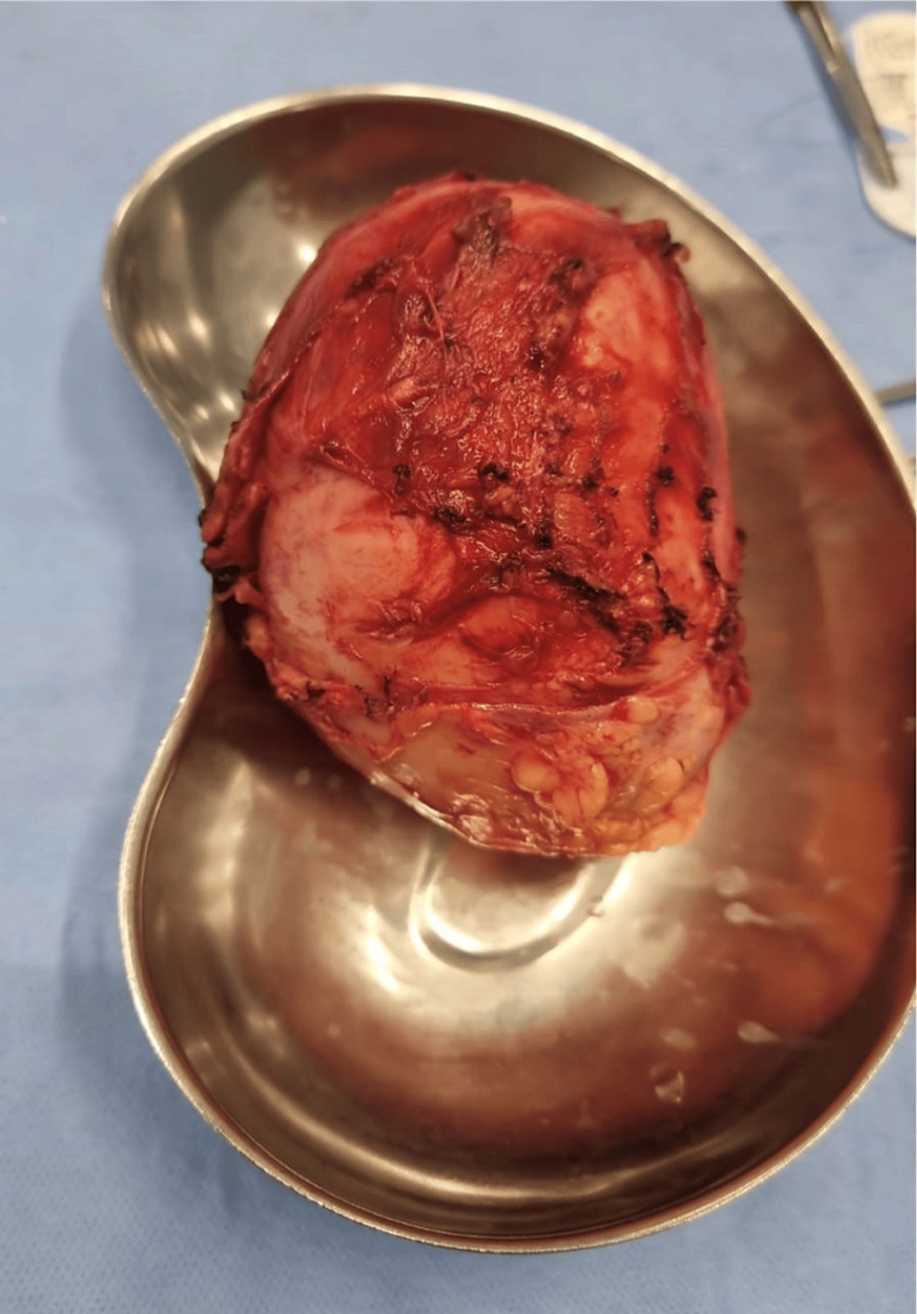
The resected retroperitoneal mass

The need for exploratory laparotomy with possible bowel resection and anastomosis was explained to the patient and family, and consent was obtained. Urology obtained separate consent for cystoscopy and ureteric catheter insertion. Laparotomy revealed a retroperitoneal mass about 11 × 12 cm in size, bounded anteriorly by the transverse colon and left kidney, superiorly by the duodenum and jejunum, laterally by the left ureter and descending colon, medially by the inferior mesenteric vein, and inferiorly by the psoas muscle. The mass was released and removed completely, and an abdominal drain was inserted (Figure 3).

**Figure 4:**
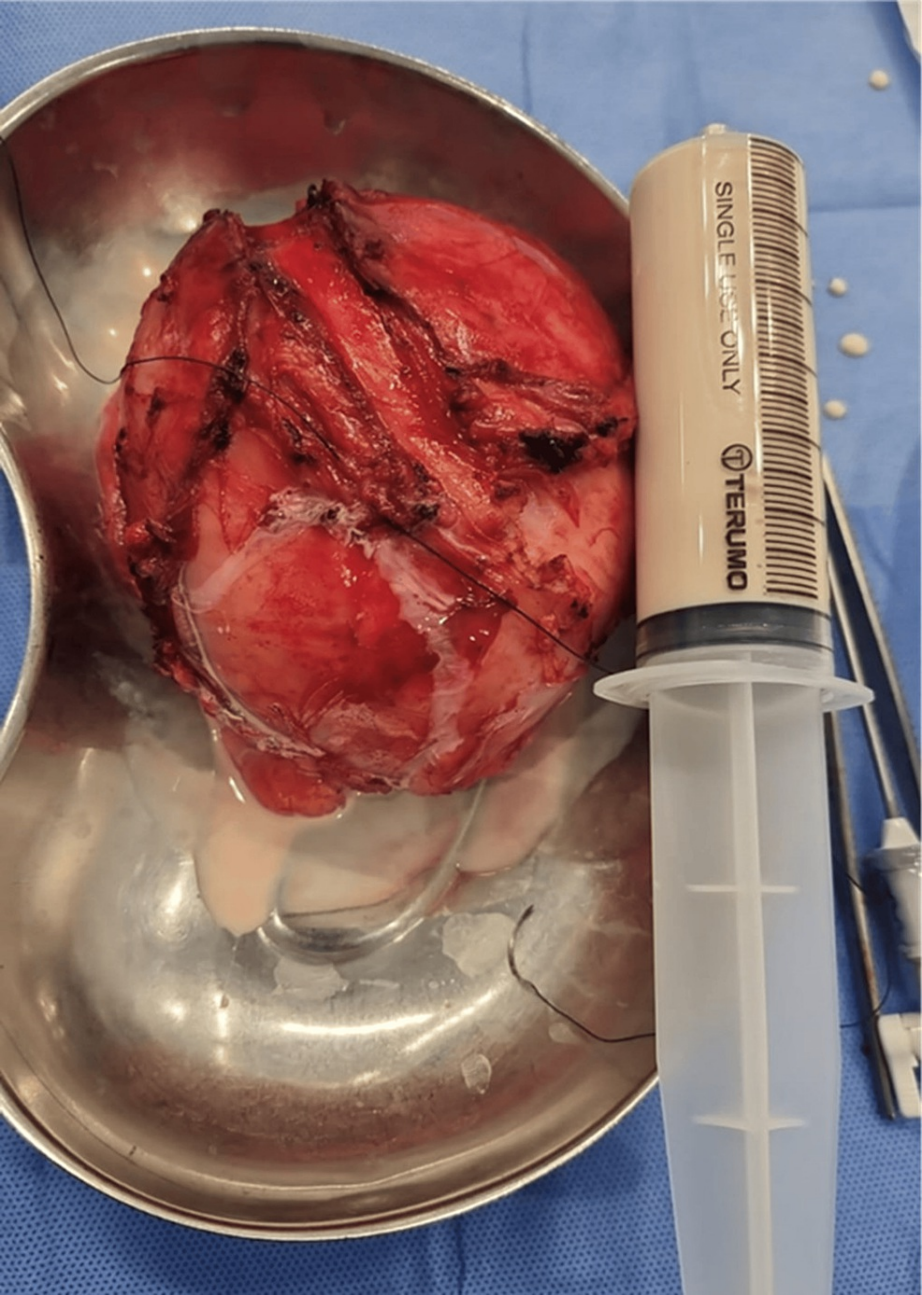
Milky fluid aspirated from the mass

Urology performed a cystoscopy and inserted a left ureteric stent. The fluid aspirated from the excised cyst was milky in appearance (Figure 4).

Histopathologic examination showed a pseudocyst with chronic inflammation and a xanthomatous reaction. No infective organism was isolated, and malignancy was ruled out. Based on these findings, our final diagnosis was retroperitoneal chyloma.

The patient’s postoperative course was uneventful. Oral feeding was started, the abdominal drain and Foley catheter were removed, and the patient was discharged in good condition 10 days after surgery. The patient was regularly followed up in the general surgery and urology clinics, and the stent was removed. After two years of following up postoperatively, no recurrence or complications were observed.

## Discussion

In a paper published in 1934, Lahey and Eckerson opined that mesenteric, omental, and retroperitoneal cysts should be grouped because they are pathologically and embryologically similar [5]. However, Kurtz et al., after a review of all cases of retroperitoneal cysts treated at their institute over 35 years, felt that retroperitoneal cysts should be considered a different entity, primarily because the outcome of surgical treatment is less satisfactory and recurrence is more common [3].

Retroperitoneal cysts can be challenging to diagnose [6]. Kurtz et al. [3] could identify no pathognomic features of retroperitoneal cysts and found the incidence to be similar in both sexes. Approximately one-third of patients with retroperitoneal cysts are asymptomatic. The symptoms that arise are due to the mass effect, which may result in obstruction or dislocation of the adjacent organs [1, 7]. Our patient had a displacement of the left kidney and hydronephrosis due to ureteric obstruction. The retroperitoneal space allows cysts to grow to a large size, and symptoms due to the mass effect may include back pain, weight loss, fever, jaundice and referred lower-limb pain [1]. Compression of pelvic vessels or ureters has been previously reported [8, 9].

Cystic lesions of the retroperitoneum may be neoplastic (e.g., cystic lymphangioma, mucinous cystadenoma, or cystic teratoma) or non-neoplastic (e.g., pancreatic pseudocyst, non-pancreatic pseudocyst, or lymphocele). Preoperative differentiation is, therefore, important. The CT features of neoplastic and non-neoplastic lesions often overlap, but lesion size, shape, and location, cyst wall presence and thickness, presence or absence of septa, calcifications, or fat, and adjacent structure involvement are findings that may provide a clue. Imaging findings, along with clinical parameters such as patient age, sex, symptoms, and clinical history, might sometimes help in preoperative diagnosis [7, 10-11]. Lymphatic cysts may be unilocular or multilocular, are lined with a single layer of flattened endothelium, and contain clear or milky fluid [10, 12].

Awareness of the imaging features of various retroperitoneal masses can help physicians narrow down the differential diagnosis [13]. According to Turkbey et al. and Prasad et al., the main feature suggestive of chyloma is a fat-fluid level within the cyst [14, 15]. In our patient, a CT scan showed the presence of fat in the superior portion of the cyst, and an MRI was able to demonstrate the fat-fluid level. Cross-sectional imaging modalities such as ultrasound and CT scan may not be able to demonstrate the fat-fluid level. Chemical shift MRI has demonstrated good diagnostic efficiency [16]. In general, for retroperitoneal masses, internal homogeneity, fat density, calcification, and cyst formation suggest benignity [17].

The treatment of choice for retroperitoneal cysts is complete excision or enucleation, although the procedure can be challenging in the retroperitoneal space due to its proximity to major vessels [3]. Smaller, more easily accessible cysts may be excised laparoscopically. Marsupialization, partial excision, or simple drainage have been tried, but recurrence is common. In our patient, complete excision was successful, and there has as yet been no recurrence. Klingenberg and Johansen have reported successful excision of a large (three-liter) retroperitoneal chylous cyst using an intercostal, retroperitoneal approach [18]. Wang et al. assessed the safety and efficacy of laparoscopic retroperitoneal resection for retroperitoneal lymphatic cysts in eight patients. The operation time was 43-88 minutes, the blood loss was 20-130 mL, and the hospital stay was three to six days. No patient suffered complications such as renal pedicle or renal pelvis injury, and no recurrences were reported. Thus, laparoscopic resection of retroperitoneal lymphatic cysts may be an effective option [19].

With the increasing use of advanced imaging techniques, smaller, asymptomatic retroperitoneal cysts are likely to be detected. Whether to operate will be a difficult decision. The cyst may never grow to become symptomatic, but if it does increase in size, the surgical risks will also increase. Thus, for small, asymptomatic retroperitoneal cysts, a wait-and-watch approach may be best [20].

## Conclusions

A retroperitoneal chylous cyst is a benign lesion that can be difficult to diagnose preoperatively. Awareness of the imaging features may help the physician narrow down the differential diagnosis. A complete excision is the treatment of choice.
